# The importance of and satisfaction with sex life among breast cancer survivors in comparison with healthy female controls and women with mental depression or arterial hypertension: results from the Finnish nationwide HeSSup cohort study

**DOI:** 10.1007/s00520-019-05228-8

**Published:** 2019-12-14

**Authors:** Minna Salakari, Raija Nurminen, Lauri Sillanmäki, Liisa Pylkkänen, Sakari Suominen

**Affiliations:** 1 Department of Public Health, University of Turku, Joukahaisenkatu 3-5, 20520 Turku, Finland; 2 University of Applied Science Turku, Joukahaisenkatu 3-5, 20520 Turku, Finland; 3 Department of Occupational Health, University of Helsinki, Mannerheimintie 172, 00300 Helsinki, Finland; 4 Department of Oncology, University of Turku, Kiinamyllynkatu 4, 20520 Turku, Finland

**Keywords:** Breast cancer, Cancer survivors, Sex life, Sexuality, Sexual satisfaction, Quality of life

## Abstract

**Introduction:**

Breast cancer (BC) and its treatment is associated with several physical and psychosocial changes that may influence sexuality for years after treatment. Women with BC show significantly greater rates of sexual dysfunction than do healthy women. The purpose of the study was to evaluate how a BC diagnosis associates with women’s perceived sexuality and sexual satisfaction.

**Material and methods:**

The data of the ongoing prospective Health and Social Support (HeSSup) survey was linked with national health registries. Respondents with registry data confirmed BC (*n* = 66), mental depression (*n* = 612), arterial hypertension (*n* = 873), and healthy women (*n* = 9731) formed the study population. The importance of and satisfaction with sex life were measured by a self-report questionnaire modified from the Schover’s and colleagues’ Sexual History Form.

**Results:**

Women with BC considered sex life less important than did healthy women (*p* < 0.001). They were significantly less satisfied with their sex life than healthy women (*p* = 0.01) and women with arterial hypertension (*p* = 0.04). Living single or educational level did not explain the differences between the groups.

**Conclusions:**

BC survivors depreciate their sex life and experience dissatisfaction with it. Sexuality can be a critical issue for the quality of life of women surviving from BC, and hence, the area deserves major attention in BC survivorship care. Health care professionals should regularly include sexual functions in the assessment of BC survivors’ wellbeing.

## Introduction

Breast cancer (BC) survivors experience worse overall sexuality than the general female population [1]. Sexuality and femininity can be critical issues for women recovering from BC. Improved screening in combination with improved treatment modalities has led to earlier diagnosis and decreased mortality and, hence, to an increase in the number of women living with BC diagnosis. This again creates new challenges for health care [2–4]. BC and its treatment is often linked to several physical and psychosocial changes. Treatment-related adverse effects can also continue to influence sexuality for years [5–8].

Sexual dysfunction is highly prevalent among women diagnosed with BC [9]. BC patients experience sexual problems soon after the start of treatment, and these problems continue in follow-up [5, 9, 10]. Several changes in a women’s sexuality following BC have been identified: lack of sexual desire and interest, body satisfaction, frequency of intercourses, sexual satisfaction, arousal, orgasm, and pain associated with intercourse [11]. Women’s negative experiences also include fear of fertility loss, and feeling of being sexually unattractive [12]. Moreover, BC survivors experience significantly poorer body image than do healthy women [13], which in turn has a negative impact on their physical and psychological functioning [14, 15] and also on the comfort of their relationships [4]. The quality of a couple relationship predicts sexuality after the BC diagnosis and treatment [12].

Sexual effects have been shown to be associated with cancer-related distress, mental depression, symptom severity, overall quality of life (QoL), marital status, and education [16, 17]. Dorval et al. [18] reported already 20 years ago BC survivors not differing from controls in general QoL domains except concerning sexuality, where BC patients reported a worse situation. However, despite the new treatment modalities, impairment of sexuality still seems to be a major problem related to BC. Depressive symptoms, age, and partnership satisfaction are critical factors for sexuality in follow-up. Low satisfaction with partnership undermines sexuality among the BC survivors. Levels of the sexual problems among BC survivors seem to exceed those of women without previous or current BC in the same age range [12, 19].

Sexuality is important from the QoL perspective and it is often not gaining sufficient attention. We considered sexuality a multidimensional, bio-psycho-social concept that includes sexual life, and biological, psychological, and sociocultural characteristics. The purpose of the study was to investigate self-reported importance of and satisfaction with sex life among BC patients compared with that of women with no BC in a case–control setting nested in a prospective cohort study design.

We hypothesized that (1) the diagnosis of BC, as a serious disease, associates negatively with women’s perceived importance of sex life, and (2) women with a BC diagnosis are more dissatisfied with their sexual life than are healthy women.

## Material and methods

### Study participants

All data presented in this study are derived from the Health and Social Support (HeSSup) survey, which is an ongoing, Finnish nationwide prospective cohort study of a representative sample of the four age groups. Age groups at the beginning of the study were 20–24, 30–34, 40–44, and 50–54 years. The study commenced in 1998 when the total number of participants was 25,895, of whom 15,267 were women and formed the cohort for this study. Follow-up postal surveys have been carried out in 2003 and 2012.

All data in this study is drawn from the 2003 questionnaire completed by linkage to data of the 2012 questionnaire and the data from the Finnish Cancer Registry, Drug Purchase and Reimbursement Registry of the Social Insurance Institution, and mortality data from Statistics Finland for the years 1998–2015. The respondents, who had a registered diagnosis of BC, and no arterial hypertension (no registered purchase of anti-hypertensive medication) or any other chronic disease formed the study group (*N* = 66). However, women with BC (*n* = 9), who had reported mental depression and had registry based anti-depressive medication, were also included in this group.

There were three comparison groups for the BC group: (1) respondents who reported having suffered from mental depression and showed registry based purchase of anti-depressive medication (*N* = 612), (2) respondents who reported having arterial hypertension and showed registry based purchase of anti-hypertensive medication (*N* = 873), and (3) all respondents of corresponding age who had not reported any chronic disease, any cancer, mental depression, or arterial hypertension (*N* = 9731). All participants had to have responded to the questions regarding satisfaction with and importance of sexual life.

The total number of BC survivors after initial recovery was relatively low, as the age of participants was generally low. Respondents diagnosed with BC prior to 1998 were excluded from the study.

**Fig. 1:**
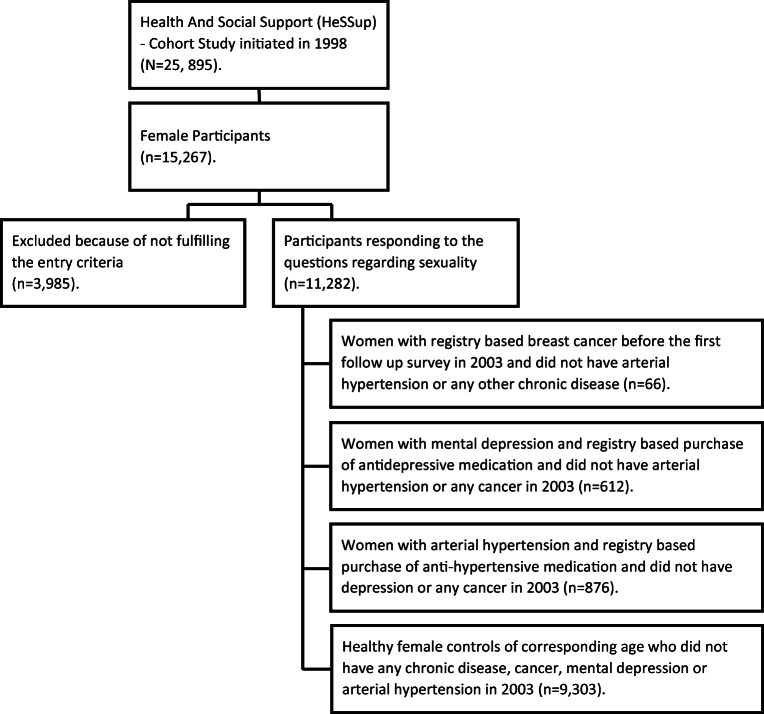
Study design

None in the comparison groups had any cancer disease. Study design is shown in Fig. 1.

### Analysis of the importance of and satisfaction with sex life

The importance of and satisfaction with sex life were assessed using a seven-point Likert scale modified from a questionnaire developed for measuring sexuality and sexual health in previous international studies [20].

The questions of sexuality were based on the Sexual History Form [20] developed from Schover’s and colleagues items. The scale is a structured, self-report questionnaire standardized according to norms from a healthy community sample and available for comparison. Reliability and validity, however, have not been formally confirmed. Two items from the Sexual History Form were selected and adapted and used in this study to provide an indicator of (1) importance of sex life and (2) overall satisfaction with sex life. The questionnaire has been translated into Finnish and Swedish including translation and back-translation and comparison with the original wording according to the translation protocol in the HeSSup-cohort study.

The format of the items was a conventional questionnaire with items presented as brief descriptive statements to which respondents rate their level of importance/satisfaction on a seven-point Likert scale. Scale interval anchors were *very important/satisfied (1)–not important at all/very dissatisfied (7).*

The respondents’ level of education was classified into four categories: no professional education; vocational course or school/apprentice contract; college; and university/other high level education.

### Statistical analyses

A logistic regression model was used to examine the associations between the study groups and level of education and relationship status. The analysis of covariance (ANCOVA) with pairwise comparison with Dunnett’s adjustment was used to determine differences between study groups.

Statistical analyses were conducted with the SAS® software v.9.4 for Windows (SAS Institute Inc., Cary, NC, USA). The level of statistical significance was *α* = 0.05.

### Ethics

The concurrent joint Ethics Committee of the University of Turku and the Turku University Central Hospital considered formal approval not necessary and stated that the study followed the ethical guidelines for good scientific practice.

## Results

### Baseline characteristics

Age distributions between study groups were not equal (Pearson’s chi-squared test, *p* < 0.001); respondents with breast cancer or arterial hypertension were older. Most of respondents lived in couple relationship (74.2%), and had vocational course/school or college education (69.6%). Respondents’ age and couple relationship are presented in Table 1.

**Table 1:** Numbers and percentages of the women by diagnosis, age group and by couple relationship according to Finnish nationwide HeSSup cohort study

Born in years	Age cohorts					Relationship status		
	1974–1978	1964–1968	1954–1958	1944–1948	All	Couple relationship	No couple relationship	All
	*N* (%)	*N* (%)	*N* (%)	*N* (%)	*N* (%)	*N* (%)	*N* (%)	*N* (%)
Breast cancer	1(1.5)	5 (7.6)	23 (34.9)	37 (56.1)	66 (100)	52 (78.8)	14 (21.2)	66 (100)
Mental depression	138 (22.6)	138 (22.6)	189 (30.9)	147 (24.0)	612 (100)	385 (63.1)	225 (36.9)	610 (100)
Arterial hypertension	27 (3.1)	70 (8.0)	216 (24.7)	563 (64.3)	876 (100)	671 (77.1)	199 (22.8)	870 (100)
Healthy controls	2980 (32.0)	2350 (25.3)	2201 (23.7)	1772 (19.1)	9303 (100)	6922 (74.6)	2353 (25.4)	9275 (100)
Total	3146 (29.0)	2563 (23.6)	2629 (24.2)	2519 (23.2)	10,857 (100)	8030 (74.2)	2791 (25.8)	10,821 (100)

The group with women suffering from mental depression lived less often in couple relationship than women in any other group (*p* < 0.001).

### Importance of sex life

The perceived importance of sex life among women with BC, mental depression, or arterial hypertension, and among healthy controls is presented in Table 2.When all age groups were combined there were no significant differences in the mean values between study groups (BC 3.76, mental depression 3.66, arterial hypertension 3.35, healthy controls 3.04) except between BC and healthy controls in the perceived importance of sex life. The BC group differed highly significantly from the group of healthy women (Dunnett *p* < 0.001). Only 7.7% of the respondents in the BC group considered sex life very important (Likert scale score 1), and 35.1% important (Likert scale score 2 or 3), while 12.1% of healthy women considered it very important and 57.3% important. Overall this difference was statistically highly significant (*p* < 0.001). Altogether 18.5% of respondents with BC did not consider sex life important (Likert scale score 6 or 7). In the group of healthy women, the corresponding result was 10.3% and in the group with mental depression 23.6% (*p* = 0.78 when BC patients were compared with patients with mental depression; Table 2).

**Table 2:** The importance of and satisfaction with sex life among women with BC, mental depression, or arterial hypertension, and among healthy female controls; age groups combined according to Finnish nationwide HeSSup cohort study

Group	How important is sex life for you? 1 (very important)–7 (not important at all)							
	1	2	3	4	5	6	7	Total
	*N* (%)	*N* (%)	*N* (%)	*N* (%)	*N* (%)	*N* (%)	*N* (%)	*N* (%)
Breast cancer	5 (7.7)	14 (21.2)	9 (13.9)	18 (27.7)	8 (12.3)	8 (12.3)	4 (6.2)	66 (0.6)
Mental depression	68 (11.2)	157 (25.7)	111 (18.2)	71 (11.6)	59 (9.8)	69 (11.3)	75 (12.3)	610 (5.6)
Arterial hypertension	100 (11.5)	220 (25.2)	207 (23.7)	144 (16.5)	79 (9.1)	68 (7.8)	55 (6.3)	873 (8.1)
Healthy controls	1121 (12.1)	3093 (33.3)	2225 (24.0)	1222 (13.2)	682 (7.4)	629 (6.9)	311 (3.4)	9283 (85.7)
Total	1294 (11.9)	3483 (32.2)	2552(23.6)	1455(13.4)	828(7.6)	774 (7.1)	445(4.1)	10,832(100)
Group	Are you satisfied with your sex life? 1 (very satisfied)–7 (very dissatisfied)							
	1	2	3	4	5	6	7	Total
	*N* (%)	*N* (%)	*N* (%)	*N* (%)	*N* (%)	*N* (%)	*N* (%)	*N* (%)
Breast cancer	6 (9.2)	12 (18.5)	10 (15.4)	16 (24.6)	10 (15.4)	7 (10.8)	4 (6.2)	65 (0.6)
Mental depression	86 (14.2)	128 (21.2)	95 (15.7)	94 (15.5)	6 (9.9)	74 (12.2)	68 (11.2)	605 (5.6)
Arterial hypertension	138 (15.9)	234 (26.9)	146 (16.8)	150 (17.2)	78 (9.0)	63 (7.2)	61 (7.0)	870 (8.1)
Healthy controls	1467 (15.9)	2729 (29.5)	1615 (17.5)	1331 (14.4)	878 (9.5)	731 (7.9)	493 (5.3)	9244 (85.7)
Total	1697 (15.7)	3103 (28.8)	1866 (17.3)	1591 (14.8)	1026 (9.5)	875 (8.1)	626 (5.8)	10,784 (100)

Living single or education did not explain the differences between the groups. However, the effect of living single on the importance of sex life was different between the groups (*p* = 0.008). Healthy women considered sex life more important than women in the other groups did. (Table 3).

**Table 3:** The association of relationship status on the importance of and perceived satisfaction with sex life in the different study groups and according to couple relationship status; age groups combined according to Finnish nationwide HeSSup cohort study. Analysis of covariance (ANOVA) with pairwise comparison with Dunnett’s adjustment

	Importance of sex life						Satisfaction with sex life					
	Couple relationship			No couple relationship			Couple relationship			No couple relationship		
	*N*	Mean	95% CI	*N*	Mean	95% CI	*N*	Mean	95% CI	*N*	Mean	95% CI
Breast cancer	52	3.63	3.23–4.04	14	4.21	3.26–5.17	52	3.62	3.18–4.05	13	4.31	3.25–5.37
Mental depression	385	3.45	3.30–3.59	223	4.05	3.81–4.29	384	3.45	3.29–3.61	219	4.07	3.82–4.33
Arterial hypertension	670	3.20	3.09–3.31	197	3.88	3.62–4.13	671	3.14	3.02–3.26	193	3.69	3.42–3.97
Healthy controls	6909	2.96*	2.93–3.00	2346	3.27	3.19–3.34	6908	2.94**	2.90–2.98	2308	3.86	3.78–3.94
Total	8016			2780			8015			2733		

*The only significant difference between BC and other groups was found in the subgroup of people living in couple relationship (BC vs. healthy controls, Dunnett-adjusted *p* = 0.02)

**The only significant difference between BC and other groups was found in the subgroup of people living in couple relationship (BC vs. healthy controls, Dunnett-adjusted *p* = 0.005)

### Satisfaction with sex life

Perceived satisfaction with sex life among women with BC, mental depression, or arterial hypertension, and among healthy female controls is presented in Table 2.

With age groups combined, there were significant differences in the mean values (BC 3.75, mental depression 3.67, arterial hypertension 3.26, healthy controls 3.17) between BC and healthy controls and between BC and arterial hypertension study groups in the perceived satisfaction with sex life.

Women with BC were significantly less satisfied with their sex life than healthy women (Dunnett *p* = 0.01) and women with arterial hypertension (Dunnett *p* = 0.04), but there were no differences compared with women with mental depression (Dunnett *p* = 0.87; Table 2). For example, only 9.2% of respondents in the BC group reported to be very satisfied (Likert scale score 1) and 33.9% satisfied (Likert scale score 2 or 3) with their sex life, while in the healthy women group corresponding results were 15.9% and 47.0%. In the group of mental depression, 14.2% of respondents were very satisfied and 36.9% satisfied with their sex life (Table 2).

When analyzed according to age group, the group with BC was statistically significantly more dissatisfied with their sex life than the respondents with arterial hypertension (*p* = 0.03) and the healthy women (*p* = 0.03, data not shown).

Living single or education did not explain the differences between the groups. According to the results, however, living alone had a different effect on satisfaction with sex life in the different groups (*p* = 0.02). In the group of healthy controls, only those with a couple relationship considered sex life more satisfactory than the others (Table 3).

## Discussion

The results obtained from this nationwide prospective cohort study describe the importance of and satisfaction with sex life in BC survivors in comparison to individuals with mental depression, arterial hypertension, and healthy women. Our results showed that there were no significant differences between women with BC, mental depression, and arterial hypertension in the perceived importance of sex life, but women with BC differed in this respect significantly from healthy women. On the other hand, the women with BC were significantly less satisfied with their sex life when compared with groups comprising of women with arterial hypertension and healthy women. Hence, our hypothesis of the dissatisfaction with sex life was shown to be true.

Sexuality and femininity have been identified among the most crucial determinants for the quality of life among women surviving cancer [9, 13, 21–23]. The negative impact of malignant disease on sexual functioning is well recognized [24]. Especially young cancer patients experience challenges in sexuality along with a lack of self-perceived attractiveness [25]. Sexual desire, importance of sex life, and satisfaction with it are reduced as generally in persons diagnosed with a life-threatening illness [13, 24].

BC patients and survivors have several sexual problems. They are dissatisfied with their sex life during treatment and follow-up [5, 9, 10]. Young women with BC have concerns regarding changes in sexuality, fertility, and body image [25]. They also report years after treatment sexual health impairments that differ significantly from those of women without a history of BC [21].

Based on our results, BC survivors considered perceived sex life quite important, but were significantly less satisfied with their sex life than were healthy women. This is likely at least partially to be related to BC treatments and various post-treatment problems, as altered body image, fear of recurrence and death, and perceived sexual attractiveness, which weaken perceived satisfaction with sex life. According to the previous studies, sexual satisfaction is associated with individual variables such as physical and psychological health status. Higher level of wellbeing is associated with increased sexual satisfaction [26, 27]. The experience of a life threatening illness, such as BC, causes also uncertainty and fears and often challenges a woman’s self-esteem, body image, and overall sexuality and relationship [12, 13].

One of our interesting findings was that BC survivors perceived their sex life less important than did healthy women, whereas the perceived importance of sex life of women with mental depression and hypertension was on the same level as among women with BC. Women with BC were also significantly less satisfied with their sex life than the healthy women or the women with arterial hypertension, which is in line with previous studies [12, 19]. This may be due to the fact that both BC and mental depression in contrast to arterial hypertension are subjectively experienced, long-term, life-threatening medical conditions.

Mental depression as a disease is associated with even 70% of increased risk of problems with sexuality. Interestingly, problems with sexuality increase the risk of mental depression by even 200% [28, 29]. Additionally, regardless of treatments, mental depression has a significant impact on overall sexuality and sexual functioning [29, 30] and sexual problems are a common side effect of many antidepressants [29].

According to our results, the status of couple relationship does not explain the differences between the study groups. Here the possibility remains that instead of the status of relationships, the quality of it is the main factor affecting the perceived importance of and the satisfaction with sex life. Low satisfaction may weaken overall sexuality among the BC survivors. Together with partnership satisfaction, the age and depressive symptoms have been reported to be critical factors [12, 19, 21].

We found that living alone had a different effect on satisfaction with sex life in different groups (Table 3). It can be stated that living in a couple relationship enhances sexual satisfaction in the healthy women’s group. In their review, del Mar Sánchez-Fuentes et al. [27] stated that sexual satisfaction is associated with individual variables such as intimate relationships and sexual response, and factors related to family relationships. Good dyadic adjustment [26] and communication [31] predict sexual satisfaction.

Sexuality and satisfaction with sex life have been seen as major factor of QoL [27, 32, 33]. On the basis of the literature review, sexual satisfaction, relationships, body image, and problems are related to QoL also among young BC patients [25]. It has been found earlier that BC survivors differed unfavorably from controls only in the sexuality domain of the QoL [18].

Ben Charif et al. [34] found that only 42.6% of young BC survivors reported being satisfied with information provided about sexuality 4 years after diagnosis. A majority of these women expressed a desire for more information on sexuality than they had received. There is also evidence that professionals are not adequately addressing the sexual information and do not sufficiently recognize needs of people with cancer [35].

Protective factors for distress include supportive social networks, such as partner and professional resources, which provide real benefit for BC survivors. Healthy overall sexuality supports QoL and the relationship between the spouses [36].

BC survivors and their partners experience sexual problems after BC treatments. Sexual problems again cause anxiety, loss of confidence, mental depression, lack of commitment, and deteriorating the relations. This suggests that not only the BC survivors but also their partners could benefit from sexual counseling [10, 24]. As cancer treatments advance and patients live longer, it is relevant to treat the impacts of BC with evidence-based interventions [37, 38]. As emphasized by Ghizzani et al. (2018), “Cancer survival has raised new needs, and caretakers have to understand the latent effects of the disease and its treatments” [37].

### Strengths and limitations

The strengths of the study are the wide source data of more than 15,000 women. The study included a low total number of BC survivors, but despite this, due to the study design, the BC group can be considered reliable and unbiased.

The other strengths of the study include the use of reliable registry data; we were not confined to only self-report. According to law, The Finnish Cancer Registry collects systematically data on all cancer cases in Finland. Hence, all BC cases in this study were confirmed. Moreover, all prescribed and over the counter medication is registered by The Finnish Drug Purchase and Imbursement Registry of the Social Insurance Institution enabling a reliable allocation of patients within groups.

The present study has, however, several limitations. At the baseline HeSSup study, the study participation rate was moderate, only 40%. Nevertheless, an analysis of non-respondents has shown that the sample is representative for the Finnish population [39]. Further, our results can only be generalized to young women with BC taking into account the age distribution of the study participants. However, the study questions concerning sexuality can be considered of prime importance for these younger age cohorts. Finally, the self-report survey design may introduce bias but previous research has shown that self-report is mostly the only way to obtain information about sensitive topics. The importance of and satisfaction with sex life were assessed using a seven-point Likert scale modified from the Sexual History Form questionnaire developed for measuring the sexuality and sexual health in previous international studies; however, except language validation, the reliability and validity of the scale have not been formally previously confirmed.

As the number of young (under 60 years) BC survivors is generally low it places this research data in a unique position. The group of BC patients in this study represents a very special group, as the majority of BC patients in the general population are over 60 years. Young BC patients may have special needs for sexual counseling, which has to be further investigated.

## Conclusions

BC survivors depreciate sex life and experience dissatisfaction with their sex life. Sexuality can be a critical issue for the quality of life of women surviving from BC. Health care professionals should regularly include sexual functioning in the evaluation of the health and wellbeing of BC survivors.

Sexuality deserves major attention in BC survivorship care. Health care professionals should regularly include sexual functions in the assessment of BC survivors’ wellbeing.

In planning of BC treatment women should be informed about the possible adverse effects of cancer treatment on sexuality, fertility, and body image. There is a need to discuss concerns of sexuality with a health care professional [24].

### Future directions and implications to health care

Research findings emphasize the development of effective, individualized psychosocial interventions for BC survivors, and their spouses to decrease cancer-related sexual impairment and promote overall wellbeing.

In connection to development and research of rehabilitation services of BC patients, sexuality should be given continued attention. Also in hospitals and outpatient clinics, development and research of patient guidance should consider sexuality aspects already during the cancer diagnosis and treatments. Healthcare professionals may need regular education on sexuality and interventions in order to support breast cancer patient and survivors.
